# Pan-immune-inflammation value and its association with all-cause and cause-specific mortality in the general population: a nationwide cohort study

**DOI:** 10.3389/fendo.2025.1534018

**Published:** 2025-04-30

**Authors:** Ye Zhang, Yong Yue, Zhengyu Sun, Pengcheng Li, Xiaoyi Wang, Gang Cheng, Hailin Huang, Zongping Li

**Affiliations:** ^1^ Department of Neurosurgery, Mianyang Central Hospital, School of Medicine, University of Electronic Science and Technology of China, Mianyang, Sichuan, China; ^2^ Division of Clinical Neuroscience, Chiba University Center for Forensic Mental Health, Chiba, Japan; ^3^ Department of Pharmacology, Chiba University Graduate School of Medicine, Chiba, Japan; ^4^ Department of Plastic and Aesthetic, Jintang First People’s Hospital, Chengdu, Sichuan, China; ^5^ The Department of Oncology, The First Affiliated Hospital of the Chengdu Medical College, Chengdu, Sichuan, China

**Keywords:** pan-immune-inflammation value, mortality, inflammation, biomarker, NHANES

## Abstract

**Introduction:**

The Pan-Immune-Inflammation Value (PIV) is a novel biomarker derived from counts of neutrophils, platelets, monocytes, and lymphocytes, providing a comprehensive measure of systemic immune and inflammatory status. While it has shown prognostic value in specific disease settings, its association with mortality in the general population remains unclear. This study aims to evaluate the predictive value of PIV for all-cause and cause-specific mortality, including cardiovascular, cancer, and diabetes-related deaths, within a general adult population.

**Methods:**

Data were obtained from the NHANES cohort, with 48,662 participants aged 20 and older. Participants were followed for an average of 117.44 months, with PIV quartiles calculated at baseline. Cox proportional hazard models were used to assess mortality risk across PIV quartiles, while restricted cubic spline models examined nonlinear dose-response relationships. Subgroup and sensitivity analyses further explored the robustness of PIV’s associations.

**Results:**

Higher PIV levels were significantly associated with increased risks of all-cause, cardiovascular, cancer, and diabetes mortality. Nonlinear relationships were observed between PIV and all-cause, cardiovascular, and cancer mortality, with a risk threshold at PIV values above 254.07. Subgroup analyses supported these findings, and sensitivity analyses confirmed the consistency of PIV’s prognostic value.

**Conclusion:**

Elevated PIV serves as an independent risk factor for multiple mortality outcomes in the general population. This study underscores the potential of PIV as a predictive biomarker for mortality risk, with implications for its use in clinical and epidemiological settings. Further studies are needed to confirm PIV’s clinical utility across diverse populations and conditions.

## Introduction

Inflammatory responses are fundamental to maintaining health and protecting the body from external threats. Acute inflammation is a normal physiological reaction to infections, injuries, and other external stimuli, where the immune system is activated to eliminate pathogens and promote tissue repair (1). However, chronic inflammation has been strongly linked to the development of various diseases, including cardiovascular diseases, cancer, diabetes, and metabolic disorders (2–7). Persistent inflammatory responses can result in tissue damage, disrupt homeostasis, and accelerate disease onset and progression (6).

Immune-inflammatory biomarkers (IIBs), such as neutrophils (NEUs), lymphocytes (LYMs), monocytes (MONs), and platelets (PLTs), reflect the balance between the host’s immune and inflammatory states and are critical for assessing disease conditions. Several inflammatory indices derived from CBC parameters, such as the monocyte-to-lymphocyte ratio (MLR), neutrophil-to-lymphocyte ratio (NLR), platelet-to-lymphocyte ratio (PLR), systemic inflammation response index (SIRI), lymphocyte-to-monocyte ratio (LMR), and systemic immune-inflammation index (SII), are widely used for disease risk assessment and prognosis. Multiple studies have demonstrated that NLR, PLR, and LMR are effective predictors of disease progression and prognosis across diverse conditions, including cancer, cardiovascular diseases, and inflammatory disorders (8–13). Additionally, these indices have been employed to distinguish between different types of chronic inflammatory diseases, such as Crohn’s disease, further underscoring their broad clinical utility (14). Among these, SII has emerged as a valuable marker of inflammation, showing significant prognostic value in chronic conditions such as cancer and inflammatory diseases (8, 15, 16). Research conducted on general populations has also highlighted the potential of SII in assessing systemic inflammation (17, 18).

More recently, a novel and more comprehensive immune-inflammatory index, the Pan-Immune-Inflammation Value (PIV), has been developed. PIV integrates the counts of NEUs, PLTs, MONs, and LYMs, offering a more holistic assessment of the systemic immune and inflammatory status (19). Preliminary studies suggest that PIV has greater prognostic accuracy compared to traditional IIBs such as NLR and PLR, particularly in predicting outcomes for patients with cancers such as advanced colorectal cancer, hepatocellular carcinoma, and breast cancer (19–21). Although PIV has shown promise in predicting outcomes for cancer patients, its association with overall and cause-specific mortality in the general population remains understudied. Therefore, this study aims to evaluate the relationship between PIV and mortality rates in the U.S. population, with the goal of determining its potential as a prognostic marker and providing valuable insights to inform public health strategies.

## Methods

### Data source and study population

This study employed a prospective cohort design, with all data drawn from the NHANES database. NHANES, administered by the National Center for Health Statistics (NCHS), uses a multistage, stratified, and subgroup probability sampling method to select a representative sample of the American population. Its objective is to evaluate the health and nutritional status of adults and children in the United States (22). The survey’s original protocol underwent a comprehensive ethical review and was approved by the CDC’s Institutional Review Board. Informed consent was obtained from all participants, who signed consent forms prior to their participation (23) Additional details regarding the study are accessible online: www.cdc.gov/nchs/nhanes/irba98.htm.

**Figure 1:**
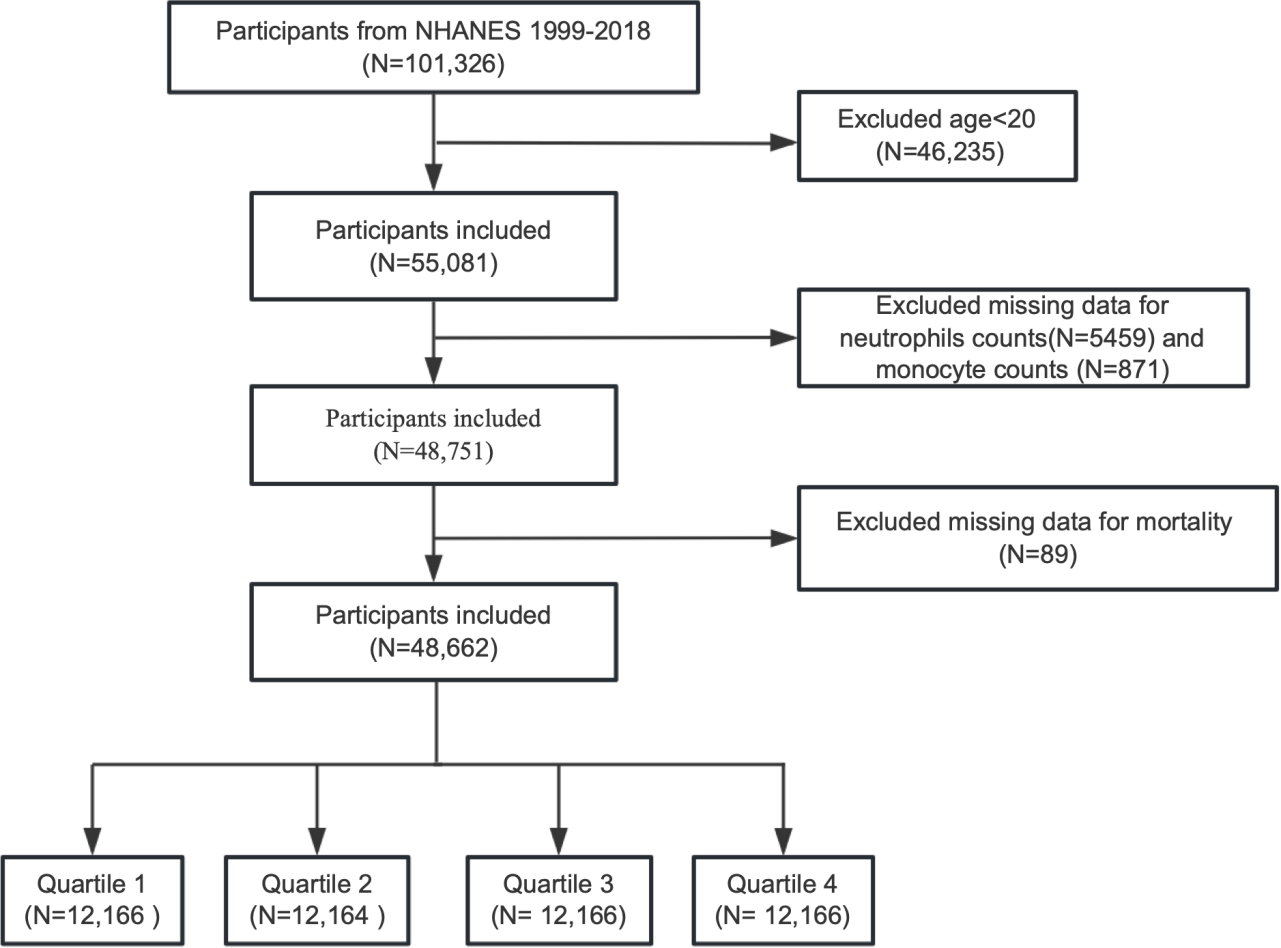
Flow chart depicting the incision and exclusion of participants from NHANES 1999-2018.

We enrolled a total of 101,326 participants from NHANES, covering data of ten circles from 1999 to 2018 in this research. Participants younger than 20 years old and those missing data on neutrophil counts, monocyte counts, or mortality information were excluded. The process of participant selection is depicted in **Figure 1**.

### Definition of CBC-derived inflammatory indices

The complete blood count (CBC) parameters were derived using the Beckman Coulter method for cell counting and sizing, with an automated diluting and mixing device for sample processing. All cell counts were measured in ×10^9^/L. The inflammatory indicators were calculated using the following formulas (19, 24):


MLR=monocytes/lymphocytes;

NLR=neutrophils/lymphocytes;

PLR=platelets/lymphocytes;

SII=platelets×neutrophils/lymphocytes;

SIRI=neutrophils×monocytes/lympocytes;

PIV=neutrophils×monocytes×platelets/lymphocytes;

As all components are expressed as counts per ×10^9^/L, the units cancel out during calculation, and all indices, including PIV, are dimensionless values. Among these indices, PIV uniquely integrates four key circulating immune cells — neutrophils, monocytes, platelets, and lymphocytes — representing both innate and adaptive immunity. Compared with simpler indices such as NLR and PLR, PIV provides a more comprehensive assessment of systemic immune-inflammatory status and has been identified as a promising prognostic marker in recent studies. In this study, PIV was analyzed both as a continuous variable and as a categorical variable by dividing participants into quartiles according to their PIV levels for subsequent analyses.

### Assessment of all-cause and cause-specific mortality

The primary outcomes of interest were all-cause mortality, along with mortality due to cardiovascular disease (CVD), diabetes and cancer. Mortality information in NHANES is available via the National Death Index (NDI) death certificate records (www.cdc.gov/nchs/data-linkage/mortality_public.htm). Participant mortality status was determined by linking their data with the National Mortality Index through December 31, 2019. Disease-specific deaths were classified according to the International Classification of Diseases (ICD)-10. Cardiovascular mortality included deaths related to heart disease, cerebrovascular conditions, and/or hypertension. Specifically, heart disease mortality corresponded to codes I00-09, I11, I13, and I20-51, while cerebrovascular mortality was defined by codes I60-I69. Diabetes-related deaths were classified under codes E10-E14, and cancer-related deaths under codes C00-C97.

### Potential covariates

Sociodemographic information assessed included age, gender, race, education level, and family income-to-poverty ratio, as well as marital status. Lifestyle and health-related factors comprised body mass index (BMI), smoking, and drinking. Laboratory parameters included red blood cell (RBC) count, white blood cell (WBC) count, lymphocyte count, neutrophil count, monocyte count, platelet count, hemoglobin, aspartate transaminase (AST), alanine transaminase (ALT), total cholesterol (TC), blood urea nitrogen (BUN), uric acid, creatinine, albumin, and glycosylated hemoglobin A1c (HbA1c). Medical conditions considered were hypertension, diabetes, kidney disease, congestive heart failure(CHF), coronary heart disease(CHD),heart attack, angina pectoris, stroke, liver disease, and cancer.

### Statistical analysis

In this study, statistical analyses accounted for the sample weights, clustering, and stratification resulting from the complex multistage stratified probability design used in NHANES. All analyses adhered to CDC guidelines (http://www.cdc.gov/nchs/tutorials/default.aspx). For two circles in NHANES 1999-2002, we applied the WTMEC4YR weights, while for the remaining 8 circles in NHANES 2003-2018, the WTMEC2YR weights were used. In accordance with the analytical recommendations, we calculated sampling weights for the 1999–2018 period as 1/5 of the 1999–2002 weight or 1/10 of the 2003–2018 weight.

Baseline characteristics of all participants were presented depending on PIV quartiles. Continuous variables were expressed as weighted means (95% confidence interval, 95%CI), while categorical variables were described in terms of weighted percentages. Differences in continuous and categorical variables were analyzed using linear regression models and chi-square tests, respectively. Multivariable Cox proportional hazards model was utilized to estimate the association between PIV and both all-cause and cause-specific mortality, reported through hazard ratios (HRs) and 95%CI. Model 1 represented the non-adjusted analysis. Model 2 adjusted for age, gender, race, family income to poverty ratio, education level, and marital status. Model 3 further adjusted for BMI, albumin, ALT, AST, BUN, creatinine, HbA1c, hemoglobin, RBC, TC, and uric acid. Finally, Model 4 included all variables from Model 3, along with adjustments for drinking, smoking, hypertension, diabetes, kidney disease, CHF, CHD, angina pectoris, heart attack, stroke, liver disease, and cancer. Survival was evaluated using the Kaplan-Meier method, and HRs for all-cause and specific mortality were derived using the log-rank test. To investigate potential non-linear associations between PIV and mortality outcomes, restricted cubic spline (RCS) analyses with four knots were performed, adjusting for the same variables as in Model 4. The knots were positioned at the 5th, 35th, 65th, and 95th percentiles of PIV distribution. Four knots were placed to exclude the most extreme 5% of values, minimizing the potential influence of outliers. Non-linearity relationship was assessed via the likelihood ratio test. In cases where a nonlinear relationship was identified, a threshold effect analysis was conducted. This involved applying a two-piece Cox proportional hazards model on either side of the inflection point to assess the association between PIV and the risk of all-cause and cause-specific mortality. Subgroup analyses were carried out to identify potential effect modifications by crucial factors, including age, gender, race, education level, family income-to-poverty ratio, marital status, smoking, drinking, BMI. The diagnostic efficacy of PIV and other inflammatory indices was evaluated using receiver operating characteristic (ROC) curve analysis. To quantify their predictive accuracy, the area under the curve (AUC) was calculated, providing a comprehensive measure of their performance in distinguishing outcomes. Finally, sensitivity analyses were performed as follows: (1) repeating the Multivariable Cox proportional hazards regression on the complete dataset (33,710 participants) without multiple imputation; (2) repeating the analyses after excluding participants with cancer, cardiovascular disease, or diabetes; and (3) calculating the E-value to determine the influence of unmeasured confounders on the study’s findings (25).

The proportion of missing data for all variables was less than 10% in our study. To address potential bias from missing data, multiple imputation was performed (26, 27). A two-sided P-value of less than 0.05 was considered statistically significant. All statistical analyses were executed using R software version 4.3.2 (R Foundation for Statistical Computing) and Empower (R) version 4.2.

## Results

### Baseline population characteristics by PIV quartiles

After excluding 46,235 participants under 20 years of age, 5,459 participants with missing neutrophil counts, 871 participants with missing monocyte counts, and 89 participants with incomplete mortality information, a total of 48,662 participants were included in the final analysis. The demographic and clinical characteristics of the participants, stratified by PIV quartiles, are detailed in **Table 1**.

**Table 1:** The demographic characteristics of the study population with various PIV quartiles.

Variable	Total (N=48,662)	Q1 <164.18(N=12,166)	Q2 164.19-254.05 (N=12,164)	Q3 254.06-393.66 (N=12,166)	Q4 >393.67(N=12,166)	P-value
Age, years	47.19 (46.83 ,47.54)	46.15 (45.66 ,46.63)	46.80 (46.38 ,47.23)	47.29 (46.78 ,47.80)	48.38 (47.88 ,48.88)	<0.001
Gender (%)						0.727
Male	48.01 (47.58 ,48.45)	48.04 (46.91 ,49.18)	48.52 (47.43 ,49.61)	47.82 (46.86 ,48.78)	47.68 (46.63 ,48.72)	
Female	51.99 (51.55 ,52.42)	51.96 (50.82 ,53.09)	51.48 (50.39 ,52.57)	52.18 (51.22 ,53.14)	52.32 (51.28 ,53.37)	
Race (%)						<0.001
Mexican American	8.20 (7.19 ,9.35)	8.35 (7.29 ,9.56)	8.34 (7.25 ,9.58)	8.47 (7.40 ,9.67)	7.67 (6.59 ,8.90)	
Hispanics	5.60 (4.80 ,6.51)	5.63 (4.85 ,6.51)	5.81 (4.89 ,6.89)	5.71 (4.84 ,6.73)	5.25 (4.34 ,6.33)	
Non-Hispanic White	68.52 (66.43 ,70.55)	57.44 (54.85 ,59.99)	68.82 (66.58 ,70.98)	71.41 (69.30 ,73.44)	75.04 (72.95 ,77.01)	
Non-Hispanic Black	10.77 (9.71 ,11.93)	19.35 (17.52 ,21.31)	10.16 (9.12 ,11.30)	8.11 (7.23 ,9.09)	6.54 (5.78 ,7.38)	
Others	6.91 (6.32 ,7.54)	9.23 (8.28 ,10.28)	6.87 (6.08 ,7.75)	6.30 (5.64 ,7.04)	5.51 (4.91 ,6.18)	
Education level (%)						<0.001
Below high school	17.23 (16.36 ,18.13)	17.38 (16.30 ,18.51)	16.86 (15.77 ,18.02)	16.60 (15.48 ,17.80)	18.09 (17.03 ,19.19)	
High school	23.99 (23.22 ,24.78)	21.65 (20.50 ,22.84)	22.85 (21.67 ,24.07)	24.43 (23.44 ,25.45)	26.73 (25.51 ,27.99)	
Above high school	58.78 (57.46 ,60.09)	60.97 (59.22 ,62.70)	60.29 (58.63 ,61.92)	58.96 (57.41 ,60.50)	55.18 (53.51 ,56.84)	
Family income of poverty ratio(%)						<0.001
<1.3	21.26 (20.23 ,22.32)	21.25 (20.09 ,22.47)	19.97 (18.65 ,21.35)	21.61 (20.26 ,23.01)	22.21 (21.01 ,23.45)	
1.30-3.5	35.92 (34.94 ,36.91)	35.07 (33.52 ,36.65)	35.26 (33.86 ,36.67)	35.40 (33.95 ,36.87)	37.85 (36.57 ,39.14)	
≥3.50	42.82 (41.29 ,44.37)	43.68 (41.64 ,45.74)	44.78 (42.90 ,46.68)	43.00 (41.03 ,44.98)	39.94 (38.18 ,41.73)	
Smoking (%)						<0.001
No	54.07 (53.14 ,55.00)	60.10 (58.62 ,61.56)	56.34 (54.91 ,57.76)	52.90 (51.54 ,54.25)	47.69 (46.22 ,49.16)	
Yes	45.93 (45.00 ,46.86)	39.90 (38.44 ,41.38)	43.66 (42.24 ,45.09)	47.10 (45.75 ,48.46)	52.31 (50.84 ,53.78)	
Drinking (%)						0.004
No	22.73 (21.61 ,23.90)	24.25 (22.76 ,25.81)	22.09 (20.77 ,23.47)	22.84 (21.38 ,24.37)	21.95 (20.69 ,23.27)	
Yes	77.27 (76.10 ,78.39)	75.75 (74.19 ,77.24)	77.91 (76.53 ,79.23)	77.16 (75.63 ,78.62)	78.05 (76.73 ,79.31)	
Marital status (%)						<0.001
Single	36.07 (35.14 ,37.01)	34.86 (33.48 ,36.27)	34.37 (33.10 ,35.65)	35.72 (34.38 ,37.08)	39.17 (37.90 ,40.46)	
Married or living with a partner	63.93 (62.99 ,64.86)	65.14 (63.73 ,66.52)	65.63 (64.35 ,66.90)	64.28 (62.92 ,65.62)	60.83 (59.54 ,62.10)	
BMI, kg/m2	28.79 (28.67 ,28.92)	27.67 (27.49 ,27.84)	28.34 (28.16 ,28.52)	29.20 (29.03 ,29.38)	29.83 (29.63 ,30.02)	<0.001
RBC, 10^12^/L	4.70 (4.69 ,4.71)	4.65 (4.63 ,4.66)	4.71 (4.69 ,4.72)	4.73 (4.71 ,4.74)	4.71 (4.69 ,4.73)	<0.001
WBC,10^9^/L	7.30 (7.26 ,7.34)	5.83 (5.77 ,5.89)	6.67 (6.62 ,6.71)	7.52 (7.47 ,7.57)	9.00 (8.94 ,9.05)	<0.001
Lymphocyte,10^9^/L	2.16 (2.14 ,2.17)	2.28 (2.23 ,2.33)	2.16 (2.14 ,2.18)	2.15 (2.13 ,2.18)	2.05 (2.03 ,2.07)	<0.001
Neutrophils,10^9^/L	4.34 (4.31 ,4.38)	2.88 (2.85 ,2.90)	3.75 (3.73 ,3.78)	4.55 (4.52 ,4.58)	6.01 (5.97 ,6.06)	<0.001
Monocyte,10^9^/L	0.56 (0.56 ,0.57)	0.43 (0.42 ,0.43)	0.51 (0.51 ,0.52)	0.58 (0.58 ,0.59)	0.72 (0.71 ,0.72)	<0.001
Platelets,10^9^/L	254.70 (253.38 ,256.02)	214.94 (213.46 ,216.42)	242.07 (240.57 ,243.58)	262.86 (261.35 ,264.38)	293.96 (291.73 ,296.20)	<0.001
Hemoglobin, g/dL	14.26 (14.22 ,14.30)	14.13 (14.08 ,14.18)	14.32 (14.28 ,14.37)	14.34 (14.30 ,14.39)	14.23 (14.17 ,14.28)	<0.001
AST, mmol/L	25.12 (24.94 ,25.30)	25.87 (25.50 ,26.24)	25.03 (24.70 ,25.35)	24.75 (24.48 ,25.02)	24.92 (24.57 ,25.27)	<0.001
ALT, mmol/L	25.29 (25.03 ,25.54)	24.92 (24.50 ,25.34)	25.53 (25.07 ,25.98)	25.23 (24.85 ,25.62)	25.43 (24.78 ,26.09)	0.191
TC, mmol/L	5.07 (5.05 ,5.09)	5.02 (4.99 ,5.05)	5.09 (5.06 ,5.11)	5.09 (5.06 ,5.12)	5.08 (5.05 ,5.10)	0.001
BUN, mmol/L	4.82 (4.78 ,4.86)	4.71 (4.65 ,4.76)	4.81 (4.77 ,4.86)	4.83 (4.78 ,4.89)	4.92 (4.86 ,4.97)	<0.001
Uric acid, umol/L	320.80 (319.63 ,321.97)	312.33 (310.20 ,314.47)	319.90 (317.77 ,322.02)	322.35 (320.48 ,324.21)	327.53 (325.20 ,329.86)	<0.001
Creatinine, umol/L	77.94 (77.50 ,78.37)	77.23 (76.60 ,77.86)	77.49 (76.84 ,78.13)	77.32 (76.63 ,78.00)	79.61 (78.81 ,80.42)	<0.001
Albumin, g/L	42.68 (42.60 ,42.76)	42.92 (42.82 ,43.03)	43.01 (42.90 ,43.11)	42.74 (42.63 ,42.85)	42.10 (42.00 ,42.20)	<0.001
HbA1c (%)	5.58 (5.56 ,5.59)	5.54 (5.52 ,5.56)	5.54 (5.52 ,5.56)	5.59 (5.56 ,5.61)	5.64 (5.62 ,5.66)	<0.001
Kidney disease (%)						<0.001
No	97.59 (97.40 ,97.76)	98.02 (97.69 ,98.31)	97.97 (97.62 ,98.27)	97.56 (97.22 ,97.86)	96.85 (96.50 ,97.16)	
Yes	2.41 (2.24 ,2.60)	1.98 (1.69 ,2.31)	2.03 (1.73 ,2.38)	2.44 (2.14 ,2.78)	3.15 (2.84 ,3.50)	
CHF (%)						<0.001
No	97.55 (97.36 ,97.74)	98.20 (97.95 ,98.42)	98.25 (97.96 ,98.49)	97.53 (97.12 ,97.87)	96.32 (95.93 ,96.68)	
Yes	2.45 (2.26 ,2.64)	1.80 (1.58 ,2.05)	1.75 (1.51 ,2.04)	2.47 (2.13 ,2.88)	3.68 (3.32 ,4.07)	
CHD (%)						<0.001
No	96.44 (96.14 ,96.70)	97.37 (96.98 ,97.71)	96.71 (96.24 ,97.12)	96.40 (95.87 ,96.87)	95.37 (94.85 ,95.84)	
Yes	3.56 (3.30 ,3.86)	2.63 (2.29 ,3.02)	3.29 (2.88 ,3.76)	3.60 (3.13 ,4.13)	4.63 (4.16 ,5.15)	
Angina pectoris (%)						<0.001
No	97.51 (97.29 ,97.71)	98.11 (97.75 ,98.41)	97.61 (97.20 ,97.96)	97.45 (97.02 ,97.82)	96.94 (96.48 ,97.34)	
Yes	2.49 (2.29 ,2.71)	1.89 (1.59 ,2.25)	2.39 (2.04 ,2.80)	2.55 (2.18 ,2.98)	3.06 (2.66 ,3.52)	
Heart attack (%)						<0.001
No	96.56 (96.30 ,96.79)	97.19 (96.77 ,97.55)	97.11 (96.67 ,97.49)	96.57 (96.11 ,96.97)	95.44 (94.96 ,95.87)	
Yes	3.44 (3.21 ,3.70)	2.81 (2.45 ,3.23)	2.89 (2.51 ,3.33)	3.43 (3.03 ,3.89)	4.56 (4.13 ,5.04)	
Stroke (%)						<0.001
No	97.15 (96.94 ,97.34)	97.66 (97.26 ,98.00)	97.72 (97.35 ,98.03)	97.15 (96.72 ,97.53)	96.12 (95.68 ,96.52)	
Yes	2.85 (2.66 ,3.06)	2.34 (2.00 ,2.74)	2.28 (1.97 ,2.65)	2.85 (2.47 ,3.28)	3.88 (3.48 ,4.32)	
Liver disease (%)						0.128
No	96.45 (96.20 ,96.68)	96.04 (95.49 ,96.53)	96.47 (95.98 ,96.90)	96.81 (96.36 ,97.21)	96.42 (95.98 ,96.82)	
Yes	3.55 (3.32 ,3.80)	3.96 (3.47 ,4.51)	3.53 (3.10 ,4.02)	3.19 (2.79 ,3.64)	3.58 (3.18 ,4.02)	
Cancer (%)						<0.001
No	90.45 (90.06 ,90.82)	91.73 (91.01 ,92.39)	90.93 (90.21 ,91.61)	90.55 (89.80 ,91.24)	88.74 (87.88 ,89.55)	
Yes	9.55 (9.18 ,9.94)	8.27 (7.61 ,8.99)	9.07 (8.39 ,9.79)	9.45 (8.76 ,10.20)	11.26 (10.45 ,12.12)	
Hypertension(%)						<0.001
No	69.26 (68.46 ,70.05)	73.04 (71.85 ,74.20)	71.63 (70.40 ,72.83)	68.80 (67.56 ,70.01)	64.04 (62.85 ,65.21)	
Yes	30.74 (29.95 ,31.54)	26.96 (25.80 ,28.15)	28.37 (27.17 ,29.60)	31.20 (29.99 ,32.44)	35.96 (34.79 ,37.15)	
Diabetes (%)						<0.001
No	91.03 (90.66 ,91.39)	92.44 (91.77 ,93.06)	92.15 (91.54 ,92.72)	90.51 (89.75 ,91.23)	89.20 (88.47 ,89.89)	
Yes	8.97 (8.61 ,9.34)	7.56 (6.94 ,8.23)	7.85 (7.28 ,8.46)	9.49 (8.77 ,10.25)	10.80 (10.11 ,11.53)	
All-cause mortality (%)						<0.001
No	88.83 (88.31 ,89.34)	91.49 (90.80 ,92.14)	90.63 (89.93 ,91.29)	89.28 (88.60 ,89.92)	84.27 (83.27 ,85.22)	
Yes	11.17 (10.66 ,11.69)	8.51 (7.86 ,9.20)	9.37 (8.71 ,10.07)	10.72 (10.08 ,11.40)	15.73 (14.78 ,16.73)	
Diabetes mortality (%)						0.001
No	99.61 (99.54 ,99.67)	99.75 (99.63 ,99.83)	99.66 (99.52 ,99.77)	99.64 (99.51 ,99.73)	99.41 (99.22 ,99.55)	
Yes	0.39 (0.33 ,0.46)	0.25 (0.17 ,0.37)	0.34 (0.23 ,0.48)	0.36 (0.27 ,0.49)	0.59 (0.45 ,0.78)	
Cancer mortality (%)						<0.001
No	97.43 (97.24 ,97.60)	97.83 (97.48 ,98.13)	97.66 (97.34 ,97.95)	97.78 (97.47 ,98.05)	96.49 (96.09 ,96.86)	
Yes	2.57 (2.40 ,2.76)	2.17 (1.87 ,2.52)	2.34 (2.05 ,2.66)	2.22 (1.95 ,2.53)	3.51 (3.14 ,3.91)	
Cardiovascular mortality (%)						<0.001
No	96.67 (96.41 ,96.91)	97.56 (97.15 ,97.91)	97.34 (96.99 ,97.65)	96.68 (96.29 ,97.03)	95.21 (94.77 ,95.62)	
Yes	3.33 (3.09 ,3.59)	2.44 (2.09 ,2.85)	2.66 (2.35 ,3.01)	3.32 (2.97 ,3.71)	4.79 (4.38 ,5.23)	
Follow-up time (months)	117.44 (115.55 ,119.34)	110.65 (108.28 ,113.03)	118.66 (116.39 ,120.94)	122.41 (119.92 ,124.90)	117.20 (114.59 ,119.81)	<0.001
MLR	0.28 (0.28 ,0.29)	0.21 (0.20 ,0.21)	0.25 (0.25 ,0.25)	0.29 (0.29 ,0.29)	0.38 (0.38 ,0.39)	<0.001
NLR	2.22 (2.20 ,2.23)	1.40 (1.39 ,1.41)	1.85 (1.83 ,1.87)	2.26 (2.24 ,2.28)	3.25 (3.21 ,3.28)	<0.001
PLR	129.99 (129.08 ,130.90)	105.75 (104.68 ,106.83)	120.86 (119.65 ,122.07)	131.93 (130.60 ,133.26)	158.40 (156.77 ,160.03)	<0.001
SII	563.98 (558.31 ,569.65)	291.18 (288.34 ,294.01)	433.75 (430.09 ,437.40)	573.61 (569.11 ,578.12)	923.36 (912.94 ,933.78)	<0.001
SIRI	1.27 (1.25 ,1.28)	0.57 (0.56 ,0.57)	0.90 (0.89 ,0.91)	1.26 (1.25 ,1.27)	2.26 (2.23 ,2.28)	<0.001
PIV	327.00 (322.80 ,331.20)	116.90 (116.10 ,117.70)	208.41 (207.82 ,209.00)	316.36 (315.37 ,317.36)	640.13 (633.21 ,647.06)	<0.001

For continuous variables: survey-weighted mean (95% CI), P-value was by survey-weighted linear regression. For categorical variables: survey-weighted percentage (95% CI), P-value was by survey-weighted Chi-square test.

BMI, body mass index; RBC, red blood cell; WBC, white blood cell; AST, aspartate transaminase; ALT, glutamic-pyruvic transaminase; TC, total cholesterol; BUN, blood urea nitrogen; HBA1c, glycosylated hemoglobin A1c; CHF, congestive heart failure; CHD, coronary heart disease; MLR, monocyte-to-lymphocyte ratio; PLR, platelet-to-lymphocyte ratio; NLR, neutrophil-to-lymphocyte ratio; SII, systemic immune-inflammation index; SIRI, systemic inflammation response index; PIV, pan-immune- inflammation value.

Participants were categorized into four quartiles based on their PIV levels at enrollment: Q1 (<164.18), Q2 (164.19–254.05), Q3 (254.06–393.66), and Q4 (>393.67). The overall mean PIV value for all participants was 327.0 (95% CI: 322.8–331.2). Median PIV values for each quartile were as follows: 116.9 (95% CI: 116.1–117.7) in Q1, 208.4 (95% CI: 207.8–209.0) in Q2, 316.4 (95% CI: 315.4–317.4) in Q3, and 640.1 (95% CI: 633.2–647.1) in Q4. Additional inflammatory markers, including MLR, NLR, PLR, SII, and SIRI, demonstrated a significant upward trend across the PIV quartiles. The mean MLR values increased from 0.21 (95% CI: 0.20–0.21) in Q1 to 0.38 (95% CI: 0.38–0.39) in Q4. Similarly, NLR rose from 1.40 (95% CI: 1.39–1.41) in Q1 to 3.25 (95% CI: 3.21–3.28) in Q4. PLR increased from 105.75 (95% CI: 104.68–106.83) in Q1 to 158.40 (95% CI: 156.77–160.03) in Q4, and SII climbed from 291.18 (95% CI: 288.34–294.01) in Q1 to 923.36 (95% CI: 912.94–933.78) in Q4. SIRI followed a similar pattern, rising from 0.57 (95% CI: 0.56–0.57) in Q1 to 2.26 (95% CI: 2.23–2.28) in Q4.

Participants in the highest PIV quartile (Q4) were characterized by older age (mean: 48.38 years), higher BMI, and a greater prevalence of females, Non-Hispanic Whites, and individuals with lower educational attainment (below high school and high school levels). They were more likely to have lower family income-to-poverty ratios (<3.5), higher rates of smoking and drinking, and a single marital status. Moreover, these participants exhibited elevated levels of inflammatory markers (MLR, NLR, PLR, SII, and SIRI) and adverse metabolic indicators, including elevated BUN, creatinine, uric acid, and HbA1c. Comorbidities such as kidney disease, cancer, hypertension, diabetes, and cardiovascular conditions (e.g., CHF, CHD, angina pectoris, heart attack, and stroke) were more prevalent in participants in Q4 compared to those in Q1. Additionally, participants in Q4 exhibited significantly higher mortality rates, including all-cause mortality (15.73% vs. 8.51%), diabetes-related mortality (0.59% vs. 0.25%), cancer-related mortality (3.51% vs. 2.17%), and cardiovascular mortality (4.79% vs. 2.44%). The median follow-up duration was shorter in Q4 participants (117.20 months) compared to Q1 (110.65 months), likely reflecting the elevated mortality risks associated with this group.

### Relationship between PIV and all-cause and cause-specific mortality

To explore the relationship between PIV and various mortality outcomes, including all-cause, cardiovascular, cancer, and diabetes-related mortality, we developed four weighted Cox proportional hazard models, as presented in **Table 2**. For every 100-unit increase in PIV, the unadjusted hazard ratios were 1.038 (95% CI: 1.030–1.047) for all-cause mortality, 1.039 (95% CI: 1.031–1.048) for cardiovascular mortality, 1.035 (95% CI: 1.028–1.042) for cancer mortality, and 1.035 (95% CI: 1.028–1.042) for diabetes mortality. The fully adjusted hazard ratios were 1.031 (95% CI: 1.024–1.038), 1.032 (95% CI: 1.024–1.040), 1.028 (95% CI: 1.020–1.035), and 1.040 (95% CI: 1.030–1.051), respectively. Furthermore, when PIV was divided into quartiles, a clear, stepwise increase in mortality risk was observed across the quartiles, even after adjusting for confounders (p for trend < 0.05).

**Table 2:** Association between PIV and all-cause mortality and cause-specific mortality.

	Model 1		Model 2		Model 3		Model 4	P-value
	HR (95% CI)	P-value	HR (95% CI)	P-value	HR (95% CI)	P-value	HR (95% CI)	
All-cause mortality								
PIV (per 100units)	1.038 (1.030,1.047)	<0.001	1.036 (1.028,1.043)	<0.001	1.030 (1.022,1.037)	<0.001	1.031 (1.024,1.038)	<0.001
Q1	Ref	Ref	Ref	Ref	Ref	Ref	Ref	Ref
Q2	1.013 (0.920, 1.116)	0.787	0.956 (0.871,1.048)	0.336	0.961 (0.874,1.057)	0.411	0.977 (0.891,1.071)	0.614
Q3	1.116 (1.021, 1.220)	0.016	1.012 (0.932,1.099)	0.771	1.003 (0.920,1.094)	0.945	0.993 (0.910,1.083)	0.869
Q4	1.718 (1.570,1.880)	<0.001	1.423 (1.312,1.544)	<0.001	1.365 (1.253,1.486)	<0.001	1.334 (1.225,1.452)	<0.001
P for trend	<0.001		<0.001		<0.001		<0.001	
Cardiovascular mortality								
PIV (per 100units)	1.039 (1.031,1.048)	<0.001	1.037 (1.029,1.045)	<0.001	1.031 (1.022,1.039)	<0.001	1.032 (1.024,1.040)	<0.001
Q1	Ref	Ref	Ref	Ref	Ref	Ref	Ref	Ref
Q2	1.004 (0.843,1.195)	0.964	0.948 (0.804, 1.118)	0.527	0.925 (0.779,1.097)	0.370	0.934 (0.788,1.109)	0.436
Q3	1.208 (1.004, 1.452)	0.045	1.098 (0.923, 1.307)	0.291	1.038 (0.869,1.240)	0.682	1.029 (0.864,1.226)	0.746
Q4	1.827 (1.562,2.136)	<0.001	1.481 (1.273, 1.724)	<0.001	1.337 (1.145,1.562)	<0.001	1.313 (1.126,1.532)	<0.001
P for trend	<0.001		<0.001		<0.001		<0.001	
Cancer mortality								
PIV (per 100units)	1.035 (1.028,1.042)	<0.001	1.030 (1.024,1.037)	<0.001	1.028 (1.021,1.035)	<0.001	1.028 (1.020,1.035)	<0.001
Q1	Ref	Ref	Ref	Ref	Ref	Ref	Ref	Ref
Q2	0.992 (0.813,1.209)	0.933	0.939 (0.772,1.141)	0.526	0.978 (0.801,1.194)	0.826	0.985 (0.806,1.204)	0.883
Q3	0.907 (0.764, 1.076)	0.263	0.830 (0.695,0.990)	0.039	0.864 (0.719,1.039)	0.120	0.855 (0.709,1.030)	0.100
Q4	1.505 (1.257, 1.802)	<0.001	1.275 (1.072,1.517)	0.006	1.317 (1.100,1.575)	0.003	1.272 (1.066,1.519)	0.008
P for trend	<0.001		<0.001		<0.001		<0.001	
Diabetes mortality								
PIV (per 100units)	1.035 (1.028,1.042)	<0.001	1.041 (1.031,1.051)	<0.001	1.028 (1.015,1.042)	<0.001	1.040 (1.030,1.051)	<0.001
Q1	Ref	Ref	Ref	Ref	Ref	Ref	Ref	Ref
Q2	1.230 (0.705,2.146)	0.467	1.214 (0.690,2.138)	0.501	1.041 (0.581,1.862)	0.894	0.981 (0.534,1.80)	0.949
Q3	1.267 (0.801, 2.002)	0.312	1.245 (0.769,2.014)	0.372	0.983 (0.583,1.657)	0.949	0.989 (0.598,1.635)	0.965
Q4	2.181 (1.368,3.477)	0.001	2.033 (1.219, 3.391)	0.007	1.580 (0.939,2.658)	0.085	1.523 (0.900, 2.577)	0.117
P for trend	<0.001		<0.001		0.004		0.011	

Model 1: Non-adjusted.

Model 2: Adjusted for age, gender, race, family income of poverty ratio, education level, marital status.

Model 3: Adjusted for age, gender, race, family income of poverty ratio, education level, marital status, BMI, albumin, ALT, AST, BUN, creatinine, HbA1c, Hemoglobin, RBC, TC, uric acid.

Model 4: Adjusted for age, gender, race, family income of poverty ratio, education level, marital status, BMI, albumin, ALT, AST, BUN, creatinine, HbA1c, Hemoglobin, RBC, TC, uric acid, drinking, smoking, hypertension, diabetes, kidney disease, CHF, CHD, angina pectoris, heart attack, stroke, liver disease, cancer.

BMI, body mass index; RBC, red blood cell; AST, aspartate transaminase; ALT, glutamic-pyruvic transaminase; TC, total cholesterol; BUN, blood urea nitrogen; HbA1c, glycosylated hemoglobin A1c; CHF, congestive heart failure; CHD, coronary heart disease; PIV, pan-immune- inflammation value; CI, confidence interval; HR, hazard ratios.

**Figure 2:**
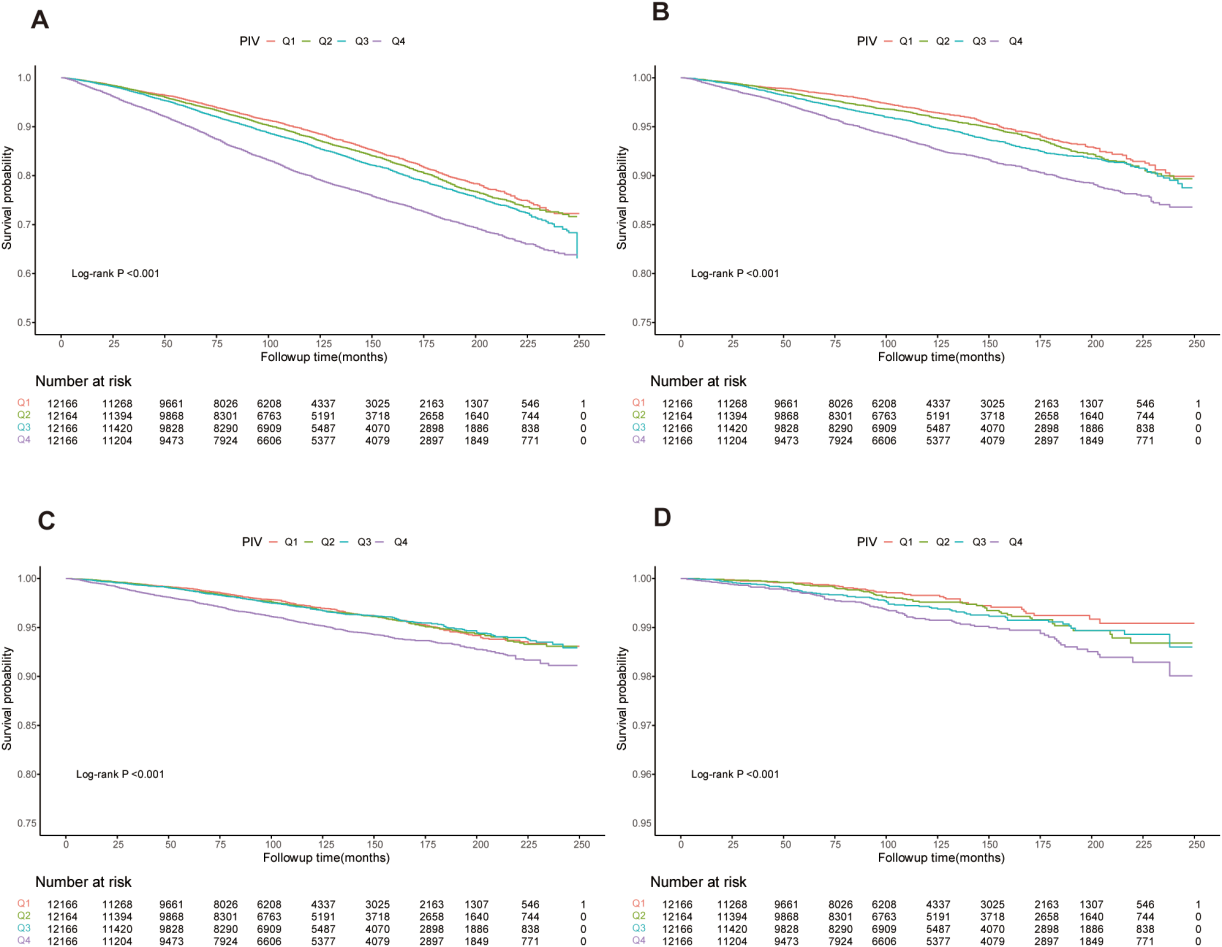
Kaplan-Meier curves showing survival rates and population numbers for us adults stratified by PIV quartiles. **(A)** All-cause mortality. **(B)** Cardiovascular mortality. **(C)** Cancer mortality. **(D)** Diabetes mortality.

Kaplan-Meier survival curves, shown in **Figure 2**, confirmed the differences in mortality rates across PIV quartiles. Significant disparities were observed in all-cause, cardiovascular, cancer, and diabetes-related mortality among the groups (log-rank test p-values < 0.001 for all).

### Nonlinear association between PIV and mortality outcomes

**Figure 3:**
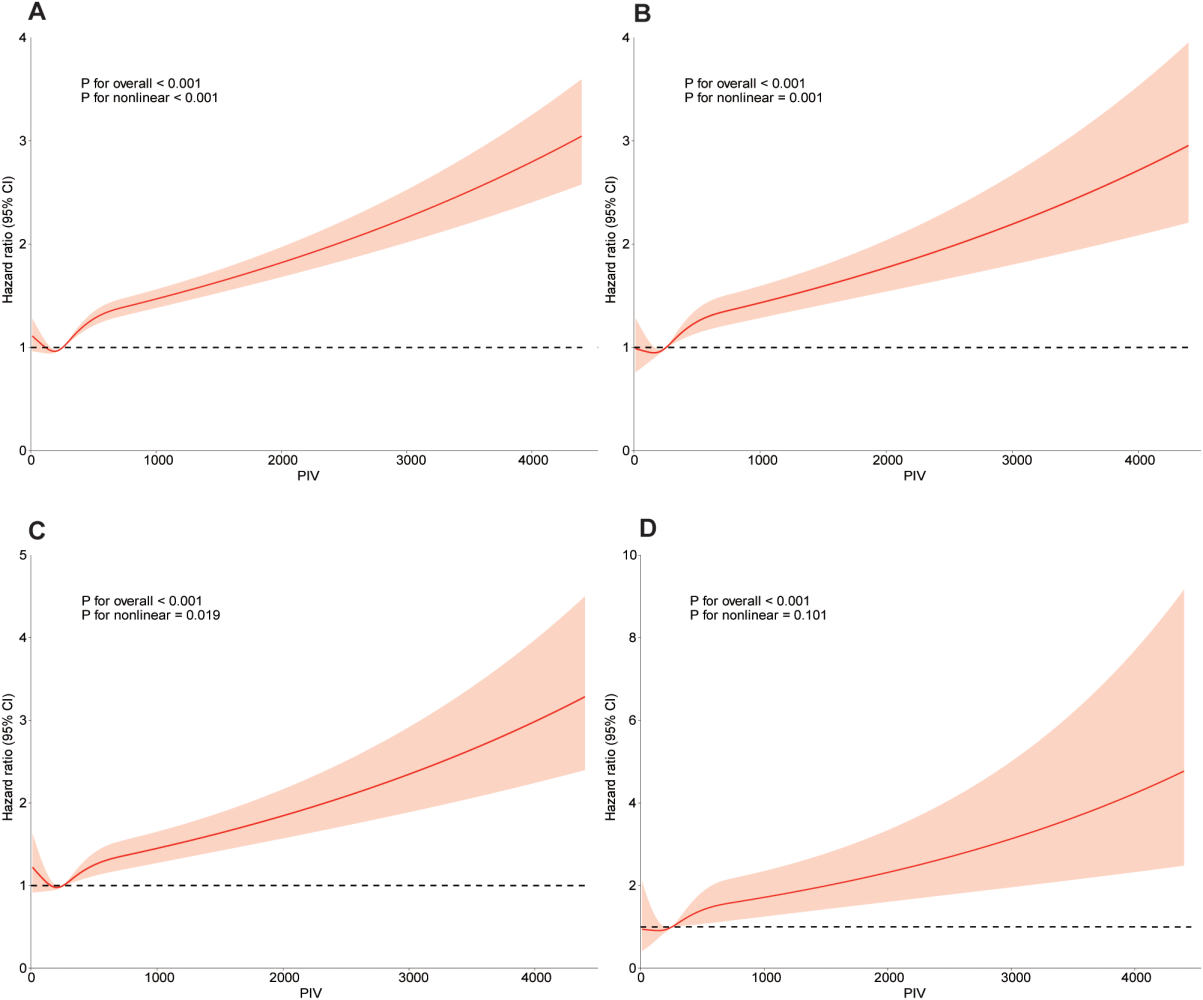
Dose-response curve of PIV and all-cause mortality and specific-mortality. A restricted cubic spline was fitted to model each curve, with 4 knots fixed at the 5th, 35th, 65th and 95th percentiles for all smooth curves. Solid lines represent the point estimates of HRs for incident all-cause mortality **(A)**, CVD mortality **(B)**, cancer mortality **(C)**, diabetes mortality **(D)**. Orange area represents their corresponding 95% Cls. Adjusted for age, gender, race, family income of poverty ratio, education level, marital status, BMI, albumin, ALT, AST, BUN, creatine, HbA1c, Hemoglobin, RBC, TC, uric acid, drinking, smoking, hypertension, diabetes, kidney disease, CHF, CHD, angina pectoris, heart attack, stroke, liver disease, cancer. PIV, pan-immune-inflammation value; BMI, body mass index; RBC, red blood cell; AST, aspartate transaminase; ALT, glutamic-pyruvic transaminase; TC, total cholesterol; BUN, blood urea nitrogen; HBA1c, glycosylated hemoglobin A1c; CHF, congestive heart failure; CHD, coronary heart disease; Cl, confidence interval; HR, hazard ratios.

To model the relationship between PIV and mortality outcomes flexibly, we used restricted cubic spline analyses. **Figure 3** illustrates significant nonlinear dose-response relationships between PIV and all-cause, cardiovascular, and cancer mortality after adjusting for covariates in Model 4 (p for nonlinearity < 0.001, 0.001, and 0.019, respectively). No significant nonlinear relationship was found between PIV and diabetes-related mortality (p for nonlinearity = 0.101).

When nonlinear relationships were identified, a threshold effect analysis was performed using a two-piece Cox proportional hazards model. For PIV values below 254.07, no significant association with all-cause, cardiovascular, or cancer mortality was observed (log-likelihood ratio test p-values = 0.995, 0.838, and 0.776, respectively). However, for PIV values of 254.07 or higher, a positive association with increased risk of all-cause, cardiovascular, and cancer mortality was evident (log-likelihood ratio test p-values < 0.001 for all), as detailed in **Supplementary Table S1**.

### Subgroup analysis

Subgroup analyses were performed to determine the association between PIV and both all-cause and cause-specific mortality, stratifying by variables including age, gender, race, family income-to-poverty ratio, marital status, education level, smoking, drinking, and BMI. Across most subgroups, PIV was consistently linked with a significantly higher risk of both all-cause and cause-specific mortality, as shown in **Table 3**. However, the interaction analysis produced nuanced results. While a significant association with all-cause and cardiovascular mortality was observed across all subgroups, except for the gender subgroup, the association for cancer mortality was significant only in the subgroups defined by race, family income-to-poverty ratio, drinking, and BMI. For diabetes mortality, significant associations were found in subgroups based on race, family income-to-poverty ratio, and drinking.

**Table 3:** Subgroup analysis of the associations between PIV (per 100units) and all-cause and cause-specific mortality.

Variables	All-cause mortality			Cardiovascular mortality			Cancer mortality			Diabetes mortality		
	HR (95%CI)	P for value	P for interaction	HR (95%CI)	P for value	P for interaction	HR (95%CI)	P for value	P for interaction	HR (95%CI)	P for value	P for interaction
Age			0.008			0.007			0.805			0.101
<60	1.05 (1.03,1.06)	<0.001		1.07 (1.04,1.09)	<0.001		1.02(0.98,1.06)	0.279		1.08 (1.03,1.13)	0.002	
≥60	1.03 (1.02,1.03)	<0.001		1.03 (1.02,1.03)	<0.001		1.03(1.02,1.03)	<0.001		1.03 (1.02,1.04)	<0.001	
Gender			0.032			0.137			0.143			0.615
Male	1.03 (1.03,1.03)	<0.001		1.03 (1.02,1.03)	<0.001		1.03(1.02,1.03)	<0.001		1.03 (1.02,1.04)	<0.001	
Female	1.04 (1.03,1.04)	<0.001		1.04 (1.03,1.05)	<0.001		1.01(0.99,1.04)	0.351		1.04 (1.01,1.07)	0.002	
Race			<0.001			<0.001			0.008			0.006
Mexican American	1.02 (1.02,1.03)	<0.001		1.02 (1.01,1.04)			1.03(1.02,1.04)	<0.001		1.02 (0.99,1.05)	0.152	
Hispanics	1.05 (1.01,1.09)	0.019		1.06 (0.99,1.13)			1.06(0.99,1.14)	0.099		1.10 (0.96,1.25)	0.162	
Non-Hispanic White	1.07 (1.06,1.07)	<0.001		1.07 (1.06,1.08)			1.05(1.03,1.07)	<0.001		1.09 (1.07,1.12)	<0.001	
Non-Hispanic Black	1.04 (1.02,1.06)	<0.001		1.04 (1.01,1.07)			1.04(1.01,1.08)	0.21		1.02 (0.92,1.13)	0.728	
Others	1.09 (1.06,1.12)	<0.001		1.09 (1.04,1.15)			1.11(1.06,1.17)	<0.001		1.04 (0.82,1.33)	0.742	
Family income of poverty ratio			<0.001			<0.001			0.047			0.004
<1.3	1.03 (1.03,1.04)	<0.001		1.04 (1.03,1.04)	<0.001		1.03(1.01,1.04)	<0.001		1.04 (1.02,1.06)	<0.001	
1.3-3.5	1.03 (1.02,1.03)	<0.001		1.03 (1.02,1.03)	<0.001		1.03(1.02,1.03)	<0.001		1.03 (1.00,1.05)	0.026	
≥3.5	1.08 (1.07,1.10)	<0.001		1.08 (1.06,1.11)	<0.001		1.07(1.04,1.09)	<0.001		1.13 (1.08,1.17)	<0.001	
Marital status			<0.001			<0.001			0.509			0.113
Single	1.03 (1.02,1.03)	<0.001		1.03 (1.02,1.03)	<0.001		1.03(1.02,1.03)	<0.001		1.03 (1.01,1.04)	<0.001	
Married or living with partner	1.04 (1.04,1.05)	<0.001		1.04 (1.04,1.05)	<0.001		1.03(1.02,1.05)	<0.001		1.05 (1.03,01.07)	<0.001	
Education level			<0.001			<0.001			0.321			0.481
Under high school	1.03 (1.03,1.04)	<0.001		1.03 (1.02,1.04)	<0.001		1.03(1.01,1.04)	<0.001		1.04 (1.01,1.06)	0.006	
High school	1.06 (1.05,1.07)	<0.001		1.07 (1.05,1.09)	<0.001		1.05(1.03,1.08)	<0.001		1.07 (1.02,1.13)	0.008	
Above high school	1.06 (1.05,1.07)	<0.001		1.03 (1.03,1.04)	<0.001		1.03(1.02,1.04)	<0.001		1.03 (1.02,1.05)	<0.001	
Smoking			<0.001			<0.001			0.203			0.148
No	1.03 (1.02,1.03)	<0.001		1.03 (1.02,1.03)	<0.001		1.03(1.02,1.04)	<0.001		1.03 (1.01,1.05)	0.001	
Yes	1.04 (1.04,1.04)	<0.001		1.04 (1.03,1.05)	<0.001		1.03(1.02,1.04)	<0.001		1.05 (1.03,1.06)	<0.001	
Drinking			<0.001			<0.001			0.003			0.004
No	1.03 (1.02,1.03)	<0.001		1.02 (1.02,1.03)	<0.001		1.03(1.02,1.03)	<0.001		1.02 (1.01,1.04)	0.002	
Yes	1.07 (1.06,1.07)	<0.001		1.07 (1.06,1.08)	<0.001		1.05(1.04,1.07)	<0.001		1.08 (1.05,1.11)	<0.001	
BMI			<0.001			<0.001			<0.001			0.057
<25	1.08 (1.07,1.09)	<0.001		1.08 (1.06,1.09)	<0.001		1.07(1.05,1.09)	<0.001		1.09 (1.04,1.14)	<0.001	
25-30	1.03 (1.02,1.03)	<0.001		1.03 (1.02,1.03)	<0.001		1.03(1.02,1.03)	<0.001		1.03 (1.01,1.05)	<0.001	
≥ 30	1.06 (1.05,1.07)	<0.001		1.07 (1.05,1.08)	<0.001		1.03(1.00,1.06)	0.021		1.06 (1.02,1.11)	0.002	

BMI, body mass index; RBC, red blood cell; AST, aspartate transaminase; ALT, glutamic-pyruvic transaminase; TC, total cholesterol; BUN, blood urea nitrogen; HbA1c, glycosylated hemoglobin A1c; CHF, congestive heart failure; CHD, coronary heart disease; PIV, pan-immune- inflammation value; CI, confidence interval; HR, hazard ratios.

### ROC analysis

**Figure 4:**
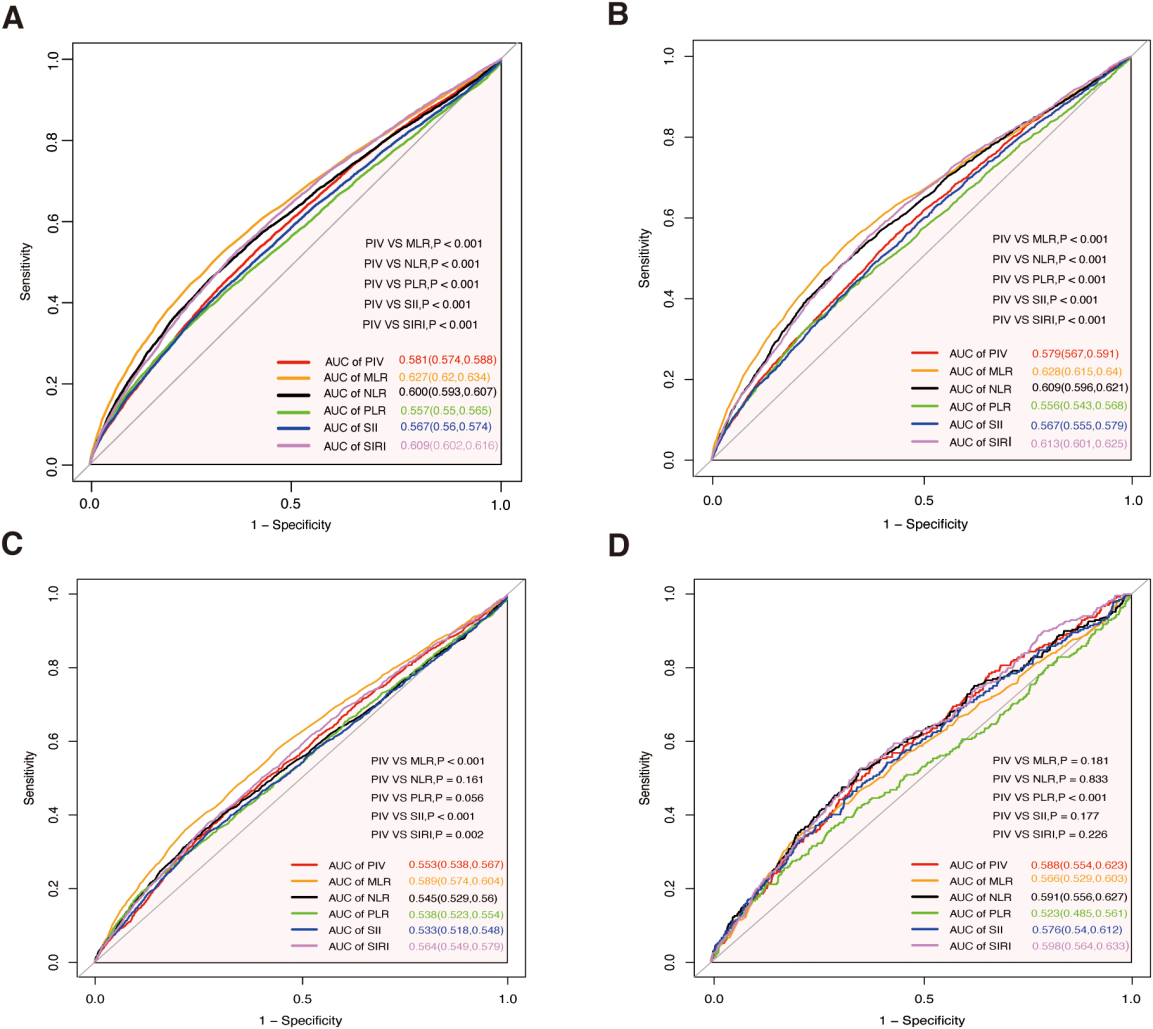
Receiver operating characteristic curves for PIV, MLR, NLR, PLR, SII, and SIRI in predicting all-cause and cause-specific mortality. **(A)** All-cause mortality. **(B)** Cardiovascular mortality. **(C)** Cancer mortality. **(D)** Diabetes mortality. MLR, monocyte-to-lymphocyte ratio; PLR, platelet-to-lymphocyte ratio; NLR, neutrophil-to-lymphocyte ratio; SII, systemic immune-inflammation index; SIRI, systemic inflammation response index; PIV, pan-immune- inflammation value; AUC, area under the curve.

ROC curve analyses (**Figure 4**) evaluated the predictive efficiency of PIV and other inflammatory markers. For all-cause mortality, PIV had an AUC of 0.581 (95% CI: 0.574–0.588), which was superior to PLR (AUC = 0.557) and SII (AUC = 0.567) (both p<0.001), but inferior to MLR (AUC = 0.627), NLR (AUC = 0.600), and SIRI (AUC = 0.609) (all p<0.001). Similar trends were observed for cardiovascular mortality, with PIV demonstrating better performance than PLR and SII (both p<0.001), but inferior to MLR, NLR, and SIRI (all p<0.001). For cancer mortality, PIV showed comparable performance to NLR and PLR, while outperforming SII (p<0.001) but being surpassed by MLR and SIRI. For diabetes-related mortality, PIV outperformed PLR (p<0.001) and was comparable to other markers (p>0.05).

### Sensitivity analysis

To further assess the stability of the PIV-mortality relationships, we performed a sensitivity analysis by excluding participants with incomplete data, as well as those with pre-existing cardiovascular disease or cancer (**Supplementary Table S2**, **Supplementary Table S3** and **Supplementary Table S4**). The results aligned with those of the primary analysis. Furthermore, based on Model 4, we calculated the E-value to determine the minimum strength of association that an unmeasured confounder would need to negate the observed PIV-mortality relationships. The E-values for PIV and all-cause mortality, cardiovascular mortality, cancer mortality, and diabetes mortality were 1.21, 1.21, 1.20, and 1.24, respectively. These E-values indicate that relatively small unmeasured confounding would be sufficient to explain the observed hazard ratios.

## Discussion

This study investigated whether the PIV could predict long-term outcomes in a general population. Our results demonstrated that PIV is significantly associated with mortality across multiple causes in this population. A high PIV level was shown to be an independent risk factor for all-cause mortality and cause-specific mortality. Additionally, PIV exhibited a nonlinear relationship with all-cause, cardiovascular, and cancer mortality, while displaying a linear association with diabetes mortality.

The PIV is a novel biomarker derived from neutrophils, platelets, monocytes, and lymphocytes, providing an integrative view of a patient’s immune and inflammatory status. Originally studied in the context of metastatic colorectal cancer, PIV has shown superior prognostic power over traditional inflammatory markers, such as NLR and PLR (19). Its simplicity, along with its ability to combine multiple immune components into a single measure, makes PIV a valuable and non-invasive tool for assessing systemic inflammation across a variety of clinical settings.

PIV has been well-established as a prognostic marker in oncology, where elevated levels are associated with worse prognosis, rapid disease progression, and therapy resistance. Researches have shown that high PIV correlates with poor survival outcomes in multiple cancers, including pancreatic (28), colorectal (29, 30), lung (31, 32), ovarian (33) esophageal (34), and breast cancers (35, 36). In newly diagnosed glioblastoma multiforme (GBM), E. Topkan et al. reported a significant association between elevated PIV levels and shorter progression-free survival (PFS) and overall survival (OS) outcomes (37). Furthermore, dynamic changes in PIV during immune checkpoint inhibitor (ICI) treatment have been linked to patient outcomes in colorectal cancer, with higher PIV levels indicating poor response and survival (38) Additionally, PIV serves as an indicator of chemotherapy resistance; for instance, in breast cancer patients undergoing neoadjuvant chemotherapy, lower PIV levels have been associated with better responses and improved survival (35). Elevated PIV levels also predict enhanced tumor progression, aiding clinicians in tailoring treatment plans and identifying patients at higher risk of recurrence. PIV not only plays a critical role in prognostic assessment but also shows potential in tumor diagnosis and recurrence monitoring. Y.T. Yang et al. highlighted that PIV has high sensitivity and specificity for diagnosing brain tumors, particularly gliomas (39). In Merkel cell carcinoma (MCC), T. Gambichler’s study confirmed that PIV levels correlate with disease stage and are independent predictors of MCC recurrence (40).

Chronic inflammation is a central factor in cardiovascular diseases, and PIV provides a comprehensive measure of inflammatory burden in conditions such as ST-segment elevation myocardial infarction (STEMI) and hypertension. Elevated PIV levels are predictive of both short-term and long-term mortality following STEMI, underscoring its value in risk stratification (9). Among hypertensive patients, high PIV levels have been linked to increased cardiovascular mortality due to their role in promoting thrombosis and exacerbating atherosclerosis (41). PIV’s capacity to integrate immune and inflammatory markers makes it a valuable tool for tracking disease progression and tailoring interventions in cardiovascular care.

Beyond oncology and cardiovascular disease, PIV has shown promise across a broad spectrum of conditions. In autoimmune diseases like systemic lupus erythematosus (SLE), PIV levels are significantly elevated compared to healthy controls (42). This elevation captures both inflammatory activity and immune dysregulation, which are critical in autoimmune disease pathogenesis. In rheumatoid arthritis, where chronic inflammation drives joint damage and cardiovascular complications, PIV serves as a useful index of inflammatory burden (43) Additionally, PIV is a significant nonlinear predictor of 28-day and 90-day mortality in septic patients, with higher levels correlating with increased mortality risk beyond a specific threshold (44). In critically ill patients with non-traumatic subarachnoid hemorrhage (SAH), elevated admission PIV is independently associated with increased mortality across ICU, in-hospital, 30-day, 90-day, and 1-year outcomes (45). For patients with fatty liver disease (FLD), Pan and colleagues demonstrated that PIV, alongside the SII, is closely associated with all-cause mortality, particularly highlighting its link to cardiovascular mortality (46). Jiang and colleagues further showed that PIV, rather than SII, is associated with the prevalence of NAFLD and hepatic fibrosis, particularly in individuals under 60, positioning it as a valuable marker for liver health (47). In hypertensive patients, Long and colleagues identified elevated PIV as a significant predictor of sarcopenia, especially in those with coexisting diabetes (48). Guo and colleagues reported that PIV, along with SII and SIRI, is inversely associated with cognitive performance in older adults, suggesting its potential as a biomarker for cognitive decline (49). Qiu and colleagues found that higher PIV levels are associated with increased COPD prevalence and all-cause mortality, with nonlinear relationships displaying a J-shaped association for prevalence and a U-shaped association for mortality risk (50). In the study by Tang et al (24), elevated levels of NLR, MLR, PLR, SII, SIRI, and PIV were positively associated with frailty risk in middle-aged and older adults, while lower PLR levels were inversely related. In frail individuals, all six inflammatory markers were linked to increased all-cause mortality, with MLR exhibiting the strongest predictive value. Among pre-frail individuals, elevated NLR, MLR, SII, SIRI, and PIV, alongside increased neutrophil counts, were associated with higher mortality risk, whereas higher lymphocyte counts were protective. Notably, a U-shaped relationship between NLR, MLR, SIRI, and PIV with mortality was observed in pre-frail individuals, where excessively low or high levels increased mortality risk. The predictive superiority of MLR likely arises from its ability to reflect immune senescence, as elevated monocytes indicate systemic inflammation, and reduced lymphocytes represent immune dysfunction—both critical drivers of frailty progression and mortality. Our study aligns with the findings of Tang et al., demonstrating that MLR exhibits the highest predictive value for all-cause mortality risk. However, their research didn’t extend to the investigation of other cause-specific mortality risks.

Taken together, these findings underscore the versatility and clinical relevance of PIV across diverse medical conditions, including liver disease, sarcopenia, cognitive decline, respiratory diseases, autoimmune disorders, sepsis, and others. PIV’s ability to integrate systemic inflammation and immune dysregulation highlights its value as a robust biomarker for risk assessment and disease prognosis across various populations.

These findings underscore the versatility and clinical relevance of PIV across diverse medical conditions, including liver disease, sarcopenia, cognitive decline, respiratory diseases, autoimmune disorders, sepsis, and others. PIV’s ability to integrate systemic inflammation and immune dysregulation highlights its value as a robust biomarker for risk assessment and disease prognosis across various populations.

While the precise mechanisms underlying PIV’s prognostic value in various diseases remain uncertain, several explanations are emerging. Firstly, neutrophils, once considered straightforward immune defenders, are now understood to regulate diverse processes, including tissue repair, cancer progression, autoimmunity, and chronic inflammation. Low neutrophil levels can lead to severe immunodeficiency, while their excessive activation can damage host tissues (51). In cancer, neutrophils release VEGF, IL-6, and MMPs, which promote angiogenesis, tumor growth, and metastasis (52). However, they also suppress adaptive immunity by inhibiting T-cell activity through nitric oxide and reactive oxygen species (ROS), enabling tumor immune evasion (53). In ischemic heart failure, neutrophils initially assist in cardiac repair by initiating inflammation and clearing necrotic myocardial debris, but prolonged activation may lead to chronic inflammation, impairing cardiac function (54). Secondly, platelets are known for their complex roles in both physiological and pathological conditions. Beyond hemostasis and thrombosis, platelets regulate immune responses, chronic inflammation, and disease progression. In sterile inflammation (e.g., atherosclerosis), platelets bind damage-associated molecular patterns (DAMPs), activate signaling pathways such as MAPK and NF-κB, and release potent inflammatory mediators like HMGB1 (55, 56) Additionally, they interact with bacteria, initiate immune responses, and release inflammatory mediators through Toll-like receptors (TLRs), aiding in pathogen defense (55) Platelets also play a crucial role in cancer metastasis by cloaking circulating tumor cells, promoting endothelial adhesion, and facilitating tumor invasion and metastasis (57, 58). Thirdly, monocytes play central roles in immune defense and inflammation. Classical monocytes are recruited to infection and inflammation sites via the CCL2/CCR2 pathway, releasing cytokines like TNF-α and iNOS to kill pathogens and enhance adaptive immunity. while non-classical monocytes patrol the vascular endothelium to monitor for tissue injury via the CX3CL1/CX3CR1 axis. In conditions like atherosclerosis, monocytes differentiate into foam cells, sustaining chronic inflammation and plaque formation (59). In tumors, monocytes differentiate into tumor-associated macrophages (TAMs), which promote immunosuppression and angiogenesis, allowing tumor cells to evade immune surveillance (60). Lastly, lymphocytes are pivotal in immune surveillance and inflammation. In chronic inflammation, such as atherosclerosis, lymphocytes mediate immune responses against pathogens and contribute to tissue repair, though excessive activity can exacerbate inflammation and tissue damage (61–63). Within tumors, tumor-infiltrating lymphocytes (TILs) recognize and kill cancer cells, particularly in high mutation-load cancers (64, 65). Conversely, lymphopenia—a low lymphocyte count—is linked with poor outcomes, reflecting impaired immune competence and heightened disease susceptibility (66). Overall, these mechanisms highlight PIV’s potential to capture the complex interplay between immunity and inflammation across diverse diseases.

Our findings underscore a significant association between PIV and various mortality outcomes. We observed a dose-dependent increase in the risk of all-cause, cardiovascular, cancer, and diabetes-related mortality with elevated PIV levels. Even after adjusting for potential confounders, high PIV levels remained consistently associated with increased mortality risks. Kaplan-Meier survival curves further validated these disparities across PIV quartiles, demonstrating that individuals with higher PIV indices had markedly elevated long-term mortality risks. Notably, restricted cubic spline analysis revealed nonlinear dose-response relationships between PIV and all-cause, cardiovascular, and cancer mortality. Specifically, when PIV levels were below 254.07, no significant association with mortality risk was observed, but once this threshold was exceeded, the risks rose sharply. This threshold effect suggests that while low PIV levels may have minimal impact, elevated PIV could play a critical role in disease progression. Sensitivity and subgroup analyses reinforced the robustness of these associations, underscoring PIV’s potential as a reliable prognostic marker across diverse populations.

Additionally, our findings highlight the comparative predictive efficiency of PIV against other inflammatory markers in mortality risk assessment. ROC curve analyses demonstrated that PIV provides reasonable predictive performance for all-cause, cardiovascular, cancer, and diabetes-related mortality. Notably, PIV outperformed simpler markers such as PLR and SII, emphasizing its greater utility in reflecting systemic inflammatory responses. However, its predictive capability was surpassed by more comprehensive indices, including MLR, NLR, and SIRI, which likely integrate broader aspects of inflammatory and immune dynamics. For cancer mortality, PIV exhibited comparable performance to NLR and PLR but was inferior to MLR and SIRI, suggesting that composite indices may better capture the complexity of inflammation-driven processes in cancer progression. Similarly, for diabetes-related mortality, PIV demonstrated consistent and comparable predictive performance relative to other markers, further supporting its reliability in specific contexts. Taken together, these findings highlight PIV as a practical and accessible prognostic marker with considerable potential for mortality risk stratification. However, the superior performance of MLR, NLR, and SIRI indicates that combining PIV with complementary markers could enhance predictive accuracy. Future studies should prioritize investigating the synergistic use of PIV alongside other inflammatory indices to improve risk stratification and inform clinical decision-making across diverse populations and mortality outcomes.

In this study, we conducted a comprehensive comparison of baseline characteristics between participants who were excluded and those included in the final analysis (**Supplementary Table S5**). Significant differences were observed in various demographic, clinical, and laboratory parameters, including age, race, educational attainment, family income-to-poverty ratio, smoking behavior, marital status, BMI, and laboratory measurements such as RBC count, lymphocyte count, platelet count, hemoglobin, ALT, TC, BUN, uric acid, creatinine, albumin, and HbA1c. Additionally, differences were notable in the prevalence of comorbidities, such as kidney disease, CHF, CHD, heart attack, stroke, and cancer, as well as in causes of mortality. Conversely, no significant differences were identified in gender, WBC count, neutrophil count, monocyte count, AST levels, or in the prevalence of angina pectoris, liver disease, hypertension, diabetes, and follow-up duration. Furthermore, inflammatory markers, including NLR, PLR, SII, and PIV, also showed no significant differences between the two groups. We recognize the potential for selection bias arising from these differences. To address this, we employed rigorous statistical adjustments, incorporating a variety of confounding variables into our analysis. Multiple models were constructed to validate the consistency and reliability of our findings, all of which demonstrated concordant trends. Despite the inherent limitations in sample selection, the robustness of our results underscores the credibility of our conclusions. This study provides a strong foundation for future research exploring the clinical relevance of PIV and related outcomes in diverse populations.

Our findings also have important clinical implications. First, as a composite biomarker derived from routine complete blood count (CBC) parameters, PIV is a cost-effective, readily available, and non-invasive marker that can be easily applied in daily clinical practice, including in primary care and resource-limited settings. Second, given its strong association with all-cause and cause-specific mortality, PIV may serve as an effective tool for early identification of individuals at high risk of adverse outcomes, who may benefit from targeted preventive interventions and more intensive clinical monitoring. For instance, individuals with elevated PIV levels could be prioritized for cardiovascular risk management, cancer screening, or metabolic evaluations. Third, since PIV reflects both innate and adaptive immune responses, as well as systemic inflammation, it offers a broader perspective on the overall immune-inflammatory status than traditional indices such as NLR or PLR, supporting its potential role in comprehensive risk stratification models. Furthermore, considering the dynamic nature of inflammation, repeated assessments of PIV over time may help monitor disease progression and evaluate treatment responses. Lastly, integrating PIV with other clinical information, including comorbidities, lifestyle factors, and biochemical markers, could improve personalized risk prediction and support clinical decision-making in preventive and therapeutic strategies. Further prospective and interventional studies are warranted to validate these clinical applications and to establish optimal PIV thresholds for risk stratification and clinical management.

## Strengths, limitations, and future directions

Our study, leveraging a large cohort and extensive follow-up, provided valuable insights into the association between PIV and mortality outcomes, including all-cause, cardiovascular, cancer, and diabetes-related mortality in the general population. The use of restricted cubic spline models enabled us to explore nonlinear relationships between PIV and mortality, revealing nuanced dose-response patterns and potential threshold effects.

However, several limitations warrant discussion. This study is cross-sectional in design, which inherently limits its ability to establish causal relationships between PIV and mortality outcomes. While the observed associations provide valuable insights, the lack of longitudinal data prevents us from fully elucidating the temporal dynamics and causal pathways underlying these relationships. This limitation is particularly relevant given the multifactorial nature of inflammation and its interactions with mortality risks over time. First and foremost, PIV was measured only at baseline, which restricts our ability to capture dynamic changes in inflammatory status during follow-up. Inflammation is a highly variable and dynamic process, and the absence of longitudinal PIV measurements may obscure important temporal trends or fluctuations that could further clarify its association with mortality. For instance, repeated measures of PIV could reveal patterns of sustained inflammation or fluctuations that are more predictive of adverse outcomes. Future studies should consider incorporating multiple PIV assessments at different time points to better evaluate its trajectory and time-dependent predictive value. Second, baseline data on complications and lifestyle factors were self-reported, which introduces the potential for recall bias and inaccuracies in the data. This limitation may have impacted the reliability of certain variables, particularly those related to behavioral factors or self-perceived health conditions. Future research should prioritize the use of objective, validated measures and standardized data collection protocols to minimize these biases and enhance the reliability of findings. Third, while we adjusted for a wide range of potential confounders, there is always the possibility of residual confounding from unmeasured variables. Factors such as genetic predisposition, environmental exposures, access to healthcare, and specific treatments during follow-up may have influenced our results. Addressing these unmeasured variables in future research through more comprehensive data collection and advanced statistical techniques, such as causal inference models, will be critical. Finally, the generalizability of our findings is limited by the single-cohort design and population characteristics. The results may not fully reflect the diverse inflammatory and mortality profiles present across different regions or healthcare systems.

To address these limitations, future research should focus on several key areas. First, well-designed longitudinal cohort studies with repeated PIV measurements are warranted to capture the dynamic changes in inflammatory status over time and to better elucidate the temporal relationship between PIV fluctuations and mortality outcomes. These studies should explore whether persistent elevation or changes in PIV trajectories are more predictive of adverse outcomes compared to single baseline measurements. Second, further investigation is needed to determine optimal PIV cut-off values for risk stratification in diverse populations, considering differences in age, sex, ethnicity, and comorbid conditions, to enhance its clinical applicability. Third, mechanistic studies incorporating multi-omics approaches, including transcriptomics, proteomics, and metabolomics, could provide deeper insights into the biological pathways linking PIV with systemic inflammation and disease progression. Fourth, intervention-based studies, such as randomized controlled trials, should assess whether modulating systemic inflammation to reduce PIV levels can translate into improved clinical outcomes, thereby establishing PIV not only as a prognostic biomarker but also as a potential target for therapeutic interventions. Additionally, future studies should integrate PIV with advanced analytical techniques, including artificial intelligence and machine learning models, to develop robust, individualized prediction tools that can dynamically assess risk based on PIV trajectories and other clinical parameters. Finally, large multi-center and international studies are essential to validate the generalizability of PIV and to facilitate its integration into global clinical practice guidelines.

Despite these limitations, our study highlights the significant prognostic value of PIV as a biomarker for mortality risks. It provides a robust foundation for future investigations into inflammation-based risk stratification, paving the way for large-scale, longitudinal, and multi-center studies to further elucidate the clinical utility of PIV in predicting diverse mortality outcomes.

## Conclusion

The PIV is a robust and versatile biomarker that integrates inflammation and immune status, providing valuable insights into disease progression, treatment response, and patient outcomes. Its prognostic utility has been demonstrated across various diseases, including cancer, cardiovascular conditions, autoimmune disorders, and infectious diseases. The individual contributions of neutrophils, platelets, monocytes, and lymphocytes reflect the intricate dynamics driving disease progression, underscoring the clinical relevance of PIV. As research advances, PIV holds substantial promise for personalized medicine, enabling clinicians to optimize treatment strategies, improve patient outcomes, and enhance healthcare delivery.

## Data Availability

The raw data supporting the conclusions of this article will be made available by the authors, without undue reservation.
