# Supplementary material for: Pan-immune-inflammation value and its association with all-cause and cause-specific mortality in the general population: a nationwide cohort study
Source: Front Endocrinol (Lausanne). 2025 Apr 30;16:1534018. doi: 10.3389/fendo.2025.1534018 (PMC12074934; doi:10.3389/fendo.2025.1534018)
Supplement: Supplementary file 1 [file Table1.docx]

**Table S1 Threshold effect analysis of PIV on all-cause and cause-specific mortality**

|  | HR (95%CI) | P for log likelihood ratio |
| --- | --- | --- |
| Fitting by the two-piecewise Cox proportional regression model |  |  |
| Inflection point | 254.07 |  |
| PIV < 254.07 （per 100 units increment） |  |  |
| All-cause mortality | 1.000(0.936,1.069) | 0.995 |
| Cardiovascular mortality | 0.986(0.861,1.129) | 0.838 |
| Cancer mortality | 1.018(0.902,1.149) | 0.776 |
| PIV ≥ 254.07（per 100 units increment） |  |  |
| All-cause mortality | 1.025(1.021,1.028) | <0.001 |
| Cardiovascular mortality | 1.026(1.018,1.034) | <0.001 |
| Cancer mortality | 1.023(1.017,1.030) | <0.001 |

Adjusted for age, gender, race, family income of poverty ratio, education level, marital status, BMI, albumin, ALT, AST, BUN, creatinine, HBA1c, Hemoglobin, RBC, TC, uric acid, drinking, smoking, hypertension, diabetes, kidney disease, CHF, CHD, angina pectoris, heart attack, stroke, liver disease, cancer

Abbreviation PIV: pan-immune- inflammation value; CI, confidence interval; HR, hazard ratios.
