# Supplementary material for: Pan-immune-inflammation value and its association with all-cause and cause-specific mortality in the general population: a nationwide cohort study
Source: Front Endocrinol (Lausanne). 2025 Apr 30;16:1534018. doi: 10.3389/fendo.2025.1534018 (PMC12074934; doi:10.3389/fendo.2025.1534018)
Supplement: Supplementary file 2 [file Table2.docx]

**Table S2 Association between PIV (100units) and all-cause mortality and specific-mortality with complete data**

|  | **Model 1** |  | **Model 2** |  | **Model 3** |  | **Model 4** |  |
| --- | --- | --- | --- | --- | --- | --- | --- | --- |
|  | **HR (95% CI)** | **P-value** | **HR (95% CI)** | **P-value** | **HR (95% CI)** | **P-value** | **HR (95% CI)** | **P-value** |
| **All-cause mortality** | |  |  |  |  |  |  |  |
| **PIV (per 100units)** | 1.030 (1.028, 1.033) | <0.001 | 1.030 (1.026, 1.033) | 0.789 | 1.023 (1.020, 1.027) | <0.001 | 1.025 (1.021, 1.029) | <0.001 |
| **Q1** | Ref | Ref | Ref | Ref | Ref | Ref | Ref | Ref |
| **Q2** | 1.064 (0.977, 1.160) | 0.154 | 0.990 (0.908, 1.079) | 0.818 | 0.983 (0.902, 1.072) | 0.705 | 0.991 (0.908, 1.081) | 0.835 |
| **Q3** | 1.207 (1.111, 1.311) | <0.001 | 1.106 (1.016, 1.204) | 0.020 | 1.074 (0.986, 1.169) | 0.102 | 1.055 (0.969, 1.148) | 0.219 |
| **Q4** | 1.678 (1.552, 1.814) | <0.001 | 1.444 (1.332, 1.566) | <0.001 | 1.342 (1.236, 1.456) | <0.001 | 1.297 (1.195, 1.408) | <0.001 |
| **P for trend** | <0.001 |  | <0.001 |  | <0.001 |  | <0.001 |  |
| **Cardiovascular mortality** | |  |  |  |  |  |  |  |
| **PIV (per 100units)** | 1.031 (1.026, 1.035) | <0.001 | 1.030 (1.024, 1.036) | <0.001 | 1.024 (1.017, 1.031) | <0.001 | 1.025 (1.018, 1.032) | <0.001 |
| **Q1** | Ref | Ref | Ref | Ref | Ref | Ref | Ref | Ref |
| **Q2** | 1.206 (1.030, 1.413) | 0.0200 | 11.113 (0.949, 1.305) | 0.190 | 1.084 (0.924, 1.272) | 0.324 | 1.085 (0.924, 1.273) | 0.319 |
| **Q3** | 1.454 (1.250, 1.692) | <0.001 | 1.323 (1.133, 1.545) | <0.001 | 1.247 (1.068, 1.457) | 0.005 | 1.223 (1.046, 1.428) | 0.011 |
| **Q4** | 1.930 (1.670, 2.230) | <0.001 | 1.614 (1.389, 1.875) | <0.001 | 1.458 (1.253, 1.697) | 0.003 | 1.416 (1.216, 1.648) | <0.001 |
| **P for trend** | <0.001 |  | <0.001 |  | <0.001 |  | <0.001 |  |
| **Cancer mortality** | |  |  |  |  |  |  |  |
| **PIV (per 100units)** | 1.029 (1.023, 1.035) | <0.001 | 1.028 (1.021, 1.035) | <0.001 | 1.023 (1.016, 1.031) | <0.001 | 1.025 (1.017, 1.033) | <0.001 |
| **Q1** | Ref | Ref | Ref | Ref | Ref | Ref | Ref | Ref |
| **Q2** | 0.914 (0.771, 1.084) | 0.303 | 0.876 (0.737, 1.042) | 0.134 | 0.901 (0.758, 1.071) | 0.235 | 0.907 (0.763, 1.078) | 0.268 |
| **Q3** | 0.955 (0.807, 1.129) | 0.589 | 0.912 (0.768, 1.083) | 0.293 | 0.926 (0.779, 1.100) | 0.381 | 0.909 (0.765, 1.080) | 0.280 |
| **Q4** | 1.373 (1.175, 1.605) | <0.001 | 1.251 (1.063, 1.471) | 0.007 | 1.234 (1.048, 1.453) | 0.012 | 1.181 (1.002, 1.391) | 0.047 |
| **P for trend** | <0.001 |  | <0.001 |  | <0.001 |  | 0.003 |  |
| **Diabetes mortality** | |  |  |  |  |  |  |  |
| **PIV (per 100units)** | 1.033 (1.021, 1.044) | <0.001 | 1.033 (1.021, 1.045) | <0.001 | 1.024 (1.010, 1.039) | 0.001 | 1.032 (1.017, 1.047) | <0.001 |
| **Q1** | Ref | Ref | Ref | Ref | Ref | Ref | Ref | Ref |
| **Q2** | 1.272 (0.801, 2.018) | 0.308 | 1.260 (0.791, 2.009) | 0.331 | 1.101 (0.689, 1.760) | 0.686 | 1.096 (0.685, 1.755) | 0.702 |
| **Q3** | 1.439 (0.919, 2.253) | 0.112 | 1.472 (0.932, 2.327) | 0.097 | 1.138 (0.716, 1.808) | 0.584 | 1.143 (0.719, 1.815) | 0.573 |
| **Q4** | 1.899 (1.238, 2.914) | 0.003 | 1.990 (1.279, 3.096) | 0.002 | 1.421 (0.909, 2.221) | 0.123 | 1.409 (0.901, 2.204) | 0.133 |
| **P for trend** | 0.002 |  | 0.001 |  | 0.083 |  | 0.092 |  |

Model 1: Non-adjusted

Model 2: Adjusted for age, gender, race, family income of poverty ratio, education level, marital status

Model 3: Adjusted for age, gender, race, family income of poverty ratio, education level, marital status, BMI, albumin, ALT, AST, BUN, creatinine, HBA1c, Hemoglobin, RBC, TC, uric acid

Model 4: Adjusted for age, gender, race, family income of poverty ratio, education level, marital status, BMI, albumin, ALT, AST, BUN, creatinine, HBA1c, Hemoglobin, RBC, TC, uric acid, drinking, smoking, hypertension, diabetes, kidney disease, CHF, CHD, angina pectoris, heart attack, stroke, liver disease, cancer

Abbreviation BMI：body mass index; RBC: red blood cell; AST: aspartate transaminase; ALT: glutamic-pyruvic transaminase; TC: total cholesterol; BUN: blood urea nitrogen; HBA1c: glycosylated hemoglobin A1c; CHF: congestive heart failure; CHD: coronary heart disease; PIV: pan-immune- inflammation value; CI, confidence interval; HR, hazard ratios.
