# Supplementary material for: Pan-immune-inflammation value and its association with all-cause and cause-specific mortality in the general population: a nationwide cohort study
Source: Front Endocrinol (Lausanne). 2025 Apr 30;16:1534018. doi: 10.3389/fendo.2025.1534018 (PMC12074934; doi:10.3389/fendo.2025.1534018)
Supplement: Supplementary file 5 [file Table5.docx]

**Table S5. Baseline characteristics between participants excluded and included**

| **Characteristic** | **Exclude(n=6419)** | **Include(n=48662)** | **P value** |
| --- | --- | --- | --- |
| **Age, years** | 47.02 (46.53,47.52) | 46.17 (46.01,46.34) | <0.001 |
| **Gender** |  |  | 0.263 |
| Male | 3043 (47.41%) | 23430 (48.15%) |  |
| Female | 3376 (52.59%) | 25232 (51.85%) |  |
| Race |  |  | <0.001 |
| Mexican American | 1074 (16.73%) | 8519 (17.51%) |  |
| Hispanics | 484 (7.54%) | 4038 (8.30%) |  |
| Non-Hispanic White | 2640 (41.13%) | 21664 (44.52%) |  |
| Non-Hispanic Black | 1589 (24.75%) | 9927 (20.40%) |  |
| Others | 632 (9.85%) | 4514 (9.28%) |  |
| **Education level** |  |  | <0.001 |
| Below high school | 2072 (32.28%) | 13068 (26.85% |  |
| High school | 1490 (23.21%) | 11243 (23.10%) |  |
| Above high school | 2803 (43.67%) | 24285 (49.90%) |  |
| Missing | 54(0.84%) | 66(0.14%) |  |
| **Family income of poverty ratio** |  |  | 0.007 |
| <1.3 | 1706 (26.58%) | 13720 (28.19%) |  |
| 1.30-3.5 | 2039 (31.76%) | 16963 (34.86%) |  |
| ≥3.50 | 1530 (23.84%) | 13806 (28.37%) |  |
| Missing | 1144(17.82%) | 4173 (8.58%) |  |
| **Smoking** |  |  | 0.007 |
| No | 3060 (47.67%) | 25582 (52.57%) |  |
| Yes | 2376 (37.02%) | 21474 (44.13%) |  |
| Missing | 983 (15.31%) | 1606(3.30%) |  |
| **Marital status** |  |  | <0.001 |
| Single | 2933 (45.69%) | 19105 (39.26%) |  |
| Married or living with a partner | 3273 (50.99%) | 29180 (59.96%) |  |
| Missing | 213 (3.32%) | 377(0.77%) |  |
| **BMI, kg/m2** | 27.72 (27.52 27.92) | 28.27 (28.21 28.32) | <0.001 |
| Missing | 2902  (45.21%) | 849(1.74%) |  |
| **RBC, 10^12^/L** | 4.58 (4.55,4.62) | 4.64 (4.64, 4.64) | 0.002 |
| Missing | 5636(87.8%) | 0 |  |
| **WBC,10^9^/L** | 6.95 (6.80,7.10) | 6.97 (6.95,6.99) | 0.818 |
| Missing | 5638 (87.83%) | 0 |  |
| **Lymphocyte,10^9^/L** | 2.16 (2.10,2.22) | 2.03 (2.03,2.04) | <0.001 |
| Missing | 5779 (90.03%) | 0 |  |
| **Neutrophils,10^9^/L** | 4.06 (3.96,4.15) | 4.00 (3.99,4.01) | 0.282 |
| Missing | 5459 (83.40%) | 0 |  |
| **Monocyte,10^9^/L** | 0.53 (0.52,0.55) | 0.53 (0.53,0.53) | 0.542 |
| Missing | 5779 (90.03%) | 0 |  |
| **Platelets,10^9^/L** | 264.93 (259.89,270.07) | 244.18 (243.59,244.78) | <0.001 |
| Missing | 5637 (87.82%) | 0 |  |
| **Hemoglobin, g/dL** | 13.54 (13.41,13.66) | 13.99 (13.97,14.00) | <0.001 |
| Missing | 5636(87.8%) | 0 |  |
| **AST, mmol/L** | 23.50 (22.87,24.15) | 23.43 (23.36,23.51) | 0.818 |
| Missing | 5698 (88.77%) | 1557 (3.2%) |  |
| **ALT, mmol/L** | 20.84 (20.07,21.64) | 21.69 (21.60,21.78) | 0.027 |
| Missing | 5698 (88.77%) | 1539(3.16%) |  |
| **TC, mmol/L** | 5.32 (5.25,5.40) | 4.94 (4.93,4.95) | <0.001 |
| Missing | 5239 (81.62%) | 699(1.44%) |  |
| **BUN, mmol/L** | 4.34 (4.22,4.47) | 4.49 (4.47,4.50) | 0.022 |
| Missing | 5779 (88.74%) | 1457(2.99%) |  |
| **Uric acid, umol/L** | 301.32 (294.95,307.82) | 310.52 (309.75,311.29) | 0.004 |
| Missing | 5696 (88.74%) | 1464(3.01%) |  |
| **Creatinine, umol/L** | 62.42 (60.73,64.17) | 74.79 (74.58,75.00) | <0.001 |
| Missing | 5697 (88.75%) | 1452(2.98%) |  |
| **Albumin, g/L** | 42.79 (42.42,43.17) | 42.08 (42.04,42.11) | <0.001 |
| Missing | 5696 (88.74%) | 1450 (2.98%) |  |
| **HBA1c (%)** | 5.56 (5.51,5.62) | 5.64 (5.64,5.65) | <0.001 |
| Missing | 5220 (81.32%) | 122 (0.25%) |  |
| **Kidney disease** |  |  | 0.014 |
| No | 6153 (95.86%) | 47045 (96.67%) |  |
| Yes | 238 (3.71%) | 1529 (3.14%) |  |
| Missing | 28 (0.44%) | 88 (0.18%) |  |
| **CHF** |  |  | <0.001 |
| No | 5148 (80.67%) | 45411 (93.32%) |  |
| Yes | 274 (4.27%) | 1546 (3.18%) |  |
| Missing | 997 (15.53%) | 1705 (3.5%) |  |
| **CHD** |  |  | 0.011 |
| No | 5136 (80.01%) | 44914 (92.30%) |  |
| Yes | 268 (4.18%) | 1980 (4.07%) |  |
| Missing | 1015 (15.81%) | 1768 (3.63%) |  |
| **Angina pectoris** |  |  |  |
| No | 5240 (81.63%) | 45585 (93.68%) | 0.064 |
| Yes | 180 (2.80%) | 1349 (2.87%) |  |
| Missing | 999 (15.56%) | 1728 (2.77%) |  |
| **Heart attack** |  |  | 0.02 |
| No | 5156 (80.32%) | 44949 (92.37%) |  |
| Yes | 277 (4.32%) | 2074 (4.26%) |  |
| Missing | 986 (15.36%) | 1639 (3.37%) |  |
| **Stroke** |  |  | <0.001 |
| No | 5137 (80.03%) | 45236 (92.96%) |  |
| Yes | 296 (4.61%) | 1803 (3.71%) |  |
| Missing | 986 (15.36%) | 1623 (3.34%) |  |
| **Liver disease** |  |  |  |
| No | 5252 (81082%) | 45196 (92.88%) | 0.104 |
| Yes | 185 (2.90%) | 1809 (3.72%) |  |
| Missing | 982 (15.3%) | 1657 (3.41%) |  |
| **Cancer** |  |  | <0.001 |
| No | 4824 (75.15%) | 42728 (87.81%) |  |
| Yes | 620 (9.66%) | 4322 (8.88%) |  |
| Missing | 975 (15.19%) | 1612 (3.31%) |  |
| **Hypertension** |  |  | 0.228 |
| No | 4187 (74.58%) | 31547 (64.83%) |  |
| Yes | 2172 (33.84%) | 16929 (34.79%) |  |
| Missing | 60 (0.93%) | 186 (0.38%) |  |
| **Diabetes** |  |  | 0.054 |
| No | 5681 (88.50%) | 42685 (87.72%) |  |
| Yes | 730 (11.37%) | 5943 (12.21%) |  |
| Missing | 8 (0.12%) | 34 (0.07%) |  |
| **All-cause mortality** |  |  | <0.001 |
| No | 4519 (70.40%) | 41233 (84.73%) |  |
| Yes | 1764 (27.18%) | 7429 (15.27%) |  |
| Missing | 136 (2.12%) | 0 |  |
| **Diabetes mortality** |  |  | 0.002 |
| No | 6363 (99.13%) | 48393 (99.45%) |  |
| Yes | 56 (0.87%) | 269 (0.55%) |  |
| **Cancer mortality** |  |  | <0.001 |
| No | 6037 (94.05%) | 47036 (96.66%) |  |
| Yes | 382 (5.95%) | 1626 (3.34%) |  |
| **Cardiovascular mortality** |  |  | <0.001 |
| No | 5854 (91.20%) | 46307 (95.16%) |  |
| Yes | 565 (8.80%) | 2355 (4.84%) |  |
| **Follow-up time (months)** | 90.13 (88.06,92.25) | 91.23 (90.61,91.84) | 0.25 |
| Missing | 136 (2.12%) | 0 |  |
| **MLR** | 0.25 (0.24,0.25) | 0.26 (0.26,0.26) | 0.001 |
| Missing | 5779 (90.03%) | 0 |  |
| **NLR** | 1.98 (1.80,2.19) | 1.97 (1.96,1.98) | 0.862 |
| Missing | 6330 (98.61%) | 0 |  |
| **PLR** | 120.13 (119.72,120.54) | 123.31 (119.88,126.83) | 0.088 |
| Missing | 5779 (90.03%) | 0 |  |
| **SII** | 481.28 (424.25,545.97) | 480.42 (478.08,482.78) | 0.976 |
| Missing | 6330 (98.61%) | 0 |  |
| **SIRI** | 0.99 (0.88,1.12) | 1.04 (1.03,1.05) | 0.445 |
| Missing | 6330 (98.61%) | 0 |  |
| **PIV** | 240.11 (207.03,278.47) | 254.07 (252.53,255.62) | 0.437 |
| Missing | 6330 (98.61%) | 0 |  |

Abbreviation BMI：body mass index; RBC: red blood cell; WBC: white blood cell; AST: aspartate transaminase; ALT: glutamic-pyruvic transaminase; TC：total cholesterol; BUN: blood urea nitrogen; HBA1c: glycosylated hemoglobin A1c; CHF: congestive heart failure; CHD: coronary heart disease; MLR： monocyte-to-lymphocyte ratio；PLR：platelet-to-lymphocyte ratio ；NLR：neutrophil-to-lymphocyte ratio；SII：systemic immune-inflammation index；SIRI：systemic inflammation response index；PIV: pan-immune- inflammation value.
